# Comparison of Clinical Laboratory Standards Institute and European Committee on Antimicrobial Susceptibility Testing guidelines for the interpretation of antibiotic susceptibility at a University teaching hospital in Nairobi, Kenya: a cross-sectional study

**DOI:** 10.1186/s12941-016-0135-3

**Published:** 2016-04-11

**Authors:** Ali Kassim, Geoffrey Omuse, Zul Premji, Gunturu Revathi

**Affiliations:** Department of Pathology, Aga Khan University Hospital, P.O. Box 30270, Nairobi, 00100 Kenya

**Keywords:** European Committee on Antimicrobial Susceptibility Testing (EUCAST), Clinical Laboratory Standards Institute (CLSI), Antimicrobial susceptibility testing (AST), Minimum inhibitory concentrations (MIC)

## Abstract

**Background:**

The Clinical Laboratory Standards Institute (CLSI) and the European Committee on Antimicrobial Susceptibility Testing (EUCAST) guidelines are the most popular breakpoint guidelines used in antimicrobial susceptibility testing worldwide. The EUCAST guidelines are freely available to users while CLSI is available for non-members as a package of three documents for US $500 annually. This is prohibitive for clinical microbiology laboratories in resource poor settings. We set out to compare antibiotic susceptibility determined by the two guidelines to determine whether adoption of EUCAST guidelines would significantly affect our susceptibility patterns.

**Methods:**

We reviewed minimum inhibitory concentrations (MIC) of various antibiotics routinely reported for *Escherichia coli* (*E. coli*), *Staphylococcus aureus* (*S. aureus*) and *Pseudomonas aeruginosa* (*P. aeruginosa*) isolates from an automated microbiology identification system (VITEK-2) at the Aga Khan University Hospital Nairobi’s Pathology department. These MICs were then analyzed using both CLSI 2015 and EUCAST 2015 guidelines and classified as resistant, intermediate or susceptible. We compared the susceptibility and agreement between the CLSI and EUCAST categorizations.

**Results:**

Susceptibility data from a total of 5165 *E. coli*, 1103 *S. aureus* and 532 *P. aeruginosa* isolates were included. The concordance rates of the two guidelines for *E. coli, S. aureus and P. aeruginosa* ranged from 78.2 to 100 %, 94.6 to 100 % and 89.1 to 95.5 % respectively. The kappa statistics for *E. coli* MICs revealed perfect agreement between CLSI and EUCAST for cefotaxime, ceftriaxone and trimethoprim–sulfamethoxazole, almost perfect agreement for ampicillin, ciprofloxacin, cefuroxime, gentamicin and ceftazidime, substantial agreement for meropenem, moderate agreement for cefepime and amoxicillin-clavulanate, fair agreement for nitrofurantoin and poor agreement for amikacin. For *S. aureus* the kappa statistics revealed perfect agreement for penicillin, trimethoprim–sulfamethoxazole, levofloxacin, oxacillin, linezolid and vancomycin, almost perfect agreement for clindamycin, erythromycin and tetracycline and moderate agreement for gentamicin. For *P. aeruginosa* the kappa analysis revealed moderate to almost perfect agreement for all the anti-pseudomonal antibiotics.

**Conclusion:**

The results show comparable antibiotic susceptibility patterns between CLSI and EUCAST breakpoints. Given that EUCAST guidelines are freely available, it makes it easier for laboratories in resource poor settings to have an updated and readily available reference for interpreting antibiotic susceptibilities.

## Background

Over the last two decades, there has been emergence and spread of antibiotic resistance in many bacterial clinical pathogens [1]. Categorization of minimum inhibitory concentrations (MIC) of various antibiotics in antimicrobial susceptibility testing (AST) depends on breakpoints set by various international agencies. These breakpoints affect clinical decision making by determining whether an antibiotic is reported as susceptible or not. One of the most popular guidelines used worldwide is from the Clinical and Laboratory Standards Institute (CLSI) whose interpretive cut offs for antibiotics is based on MIC distributions, pharmacokinetic–pharmacodynamic (PK-PD) properties and the mechanisms of resistance [1]. The adoption of antibiotic susceptibility guidelines in the US is regulated by the Food and Drug Administration (FDA). In 1997 various national agencies in Europe came together to form the European Committee on Antimicrobial Susceptibility Testing (EUCAST) that has since harmonized antibiotic interpretive breakpoints throughout Europe and most European countries have since switched from CLSI and their local guidelines to EUCAST guidelines [2, 3]. EUCAST bases its clinical breakpoints on epidemiological MIC cut-offs (ECOFFS) and PK-PD properties. All documents on MIC distributions and ECOFFS are freely available on the EUCAST website [4].

Polsfuss et al. compared EUCAST with CLSI in screening for extended-spectrum beta-lactamase (ESBL) producing *Enterobacteriaceae* isolates. They found no significant difference in the sensitivity of the two guidelines in the detection of ESBL-producing isolates [5]. A more recent study by Hombach et al. demonstrated significant differences in the susceptibility rates for drugs including cefepime, ceftazidime and cefotaxime in the detection of ESBLs between the CLSI 2013 and EUCAST 2013 AST guidelines. This study recommended adjustments to the clinical breakpoints to further harmonize the two guidelines [6]. Since then 2014 and 2015 guidelines for both systems have been released in an effort to harmonize the clinical breakpoints [7, 8].

CLSI guidelines have a number of disadvantages. First, it is based on annual subscriptions of US $350 for members and a cost of US $500 to non-members annually and this may be a problem for microbiology laboratories in resource poor settings. Secondly, details on the decision making process are not accessible to the public. Thirdly, the FDA has a major influence in determining official clinical breakpoints before they are adopted by CLSI and this raises major concerns on the influence of pharmaceutical industries in establishing the guidelines. CLSI has a voting committee comprising representatives of both the profession and the industry and hence the industry plays a role in the decision making process. As for EUCAST, the industry only plays a consultative role and is not allowed to finance or participate in decision making [3]. EUCAST encourages the formation of National Antimicrobial Susceptibility Testing Committees (NAC) that can send a representative to sit in its General Committee (GC) thus fostering inclusivity in the decision making process [3]. Finally, antibiotics that are not registered in the US may not be included in the CLSI guidelines.

For resource poor settings like Africa, there is need for guidelines that are accessible and affordable while still maintaining quality of reported susceptibility. Given that antibiotic susceptibility is quite variable across different settings, we set out to compare the susceptibility rates of selected antibiotics based on CLSI 2015 and EUCAST 2015 AST guidelines and the level of agreement between the two guidelines.

## Methods

The study was carried out at the Aga Khan University Hospital, Nairobi’s (AKUHN) Department of Pathology. Ethical exemption was granted by the AKUHN’s research and ethics committee (Ref 2015/REC-44) since this type of study is low risk and classified as a clinical audit. This was a retrospective study reviewing the MICs of various antimicrobials on one commonly isolated gram positive organism, *Staphylococcus aureus* (*S. aureus*), one fermenting gram negative organism, *Escherichia coli* (*E. coli*) and one non-fermenting gram negative organism, *Pseudomonas aeruginosa* (*P. aeruginosa*). MIC data for *E. coli, P. aeruginosa* and *S. aureus* isolates was collected from two Vitek 2 (version 4.01, bioMerieux, Marcy-l’Etoile, France) automated microbiology systems for the period January 2012 to December 2014. The Vitek 2 AST-P580 and AST-GN26/AST-GN83 cards were used for antibiotic susceptibility for *S. aureus* and *E. coli/P. aeruginosa* respectively. Ceftriaxone MICs were only available for the year 2014 in the AST-GN83 cards for a total of 1673 *E. coli* isolates. The data was summarized using Microsoft Excel 2013 and imported into IBM (International Business Machines, Corporation; Armonk, New York, United States of America) SPSS (Statistical Package for the Social Sciences) Version 22.0 that was used for analysis. The MICs were then analyzed using both the CLSI 2015 and EUCAST 2015 guidelines to categorize them as either susceptible, intermediate or resistant [7, 8]. The concordance rate between the two guidelines in percentage was compared. The susceptibility rates for the various antimicrobial agents were also calculated in percentages for each organism. Analysis of the extent of agreement between CLSI 2015 and EUCAST 2015 for the various antimicrobials was carried out using Cohen’s kappa statistics and graded from perfect agreement to poor agreement [9]. Cohen’s Kappa statistics determines the proportion of agreement over and above chance between two independent observations. This ranges from −1 to 1 and a p value less than 0.05 means that the agreement reported is significantly different from 0 and is not due to chance. For all inferential statistics, a p value less than 0.05 was considered statistically significant.

## Results

A total of 5165 *E. coli,* 1103 *S. aureus* and 532 *P. aeruginosa* non-duplicate isolates were included in the analysis. Of the 5165 *E. coli* MICs analyzed, there was comparable susceptibility to most antibiotics including ampicillin, amoxicillin–clavulanate, cefuroxime, cefotaxime, ceftriaxone, trimethoprim–sulfamethoxazole, ciprofloxacin, gentamicin and meropenem between the two guidelines. The concordance between the two guidelines ranged from 78.2 to 100 %. Table 1 summarizes the susceptibilities, concordance and kappa statistics between the two guidelines for *E. coli*.

**Table 1:** Susceptibilities of *E. coli* to various antibiotics, concordance and kappa statistics between CLSI 2015 and EUCAST 2015 guidelines

Antibiotic	CLSI (%); n = 5165			EUCAST (%); n = 5165			Concordance (%)	Kappa, κ (95 % CI)
	S	I	R	S	I	R		
Ampicillin	13.8	0.5	85.7	13.8	0	86.2	99.5	0.985 (0.979, 0.991)
Amox-Clav	55.8	23.8	20.4	55.8	0	44.2	78.2	0.581 (0.567, 0.595)
TMP/SMX	0	0	100	0	0	100	100	1
Nitrofurantoin	84.6	11.9	3.5	96.5	0	3.5	87.6	0.351 (0.314, 0.388)
Ciprofloxacin	57.3	0.2	42.5	55.9	1.4	42.7	98.4	0.969 (0.963, 0.975)
Cefuroxime	63.7	3.5	32.8	63.7	0	36.3	96.5	0.924 (0.914, 0.934)
Gentamicin	78.5	0.2	21.3	78.1	0.4	21.5	99.5	0.979 (0.973, 0.985)
Amikacin	99.3	0.3	0.4	90.5	8.8	0.7	91.1	0.112 (0.079, 0.145)
Cefotaxime	69.7	0.4	29.9	69.7	0.4	29.9	100	1
Ceftazidime	76.3	0.9	22.8	71.5	4.8	23.7	93.7	0.859 (0.843, 0.85)
Ceftriaxone	67.7	0.1	32.2	67.7	0.1	32.2	100	1
Cefepime	80.5	10.0	9.5	72.9	9.8	17.3	84.9	0.600 (0.578, 0.622)
Meropenem	99.7	0.1	0.2	99.8	0.1	0.1	99.7	0.724 (0.573, 0.875)

*Amox-Clav* amoxicillin–clavulanate, *TMP/SMX* trimethoprim–sulfamethoxazole, *S* susceptible, *I* intermediate, *R* resistant

The *E. coli* susceptibility patterns achieved after analysis using EUCAST 2015 and CLSI 2015 guidelines are similar except for amoxicillin–clavulanate, amikacin and nitrofurantoin. Analysis year by year did not show any difference in the trends and overall susceptibilities. The kappa analysis revealed perfect agreement for ceftriaxone, cefotaxime and trimethoprim–sulfamethoxazole with kappa (κ) of 1 (p < 0.000). An almost perfect agreement was noted with ampicillin κ = 0.985 (95 % CI 0.979, 0.991), p < 0.000, ciprofloxacin κ = 0.969 (95 % CI 0.963, 0.975), p < 0.000, cefuroxime κ = 0.924 (95 % CI 0.914, 0.934), p < 0.000, ceftazidime κ = 0.859 (95 % CI 0.843, 0.85), p < 0.000 and gentamicin κ = 0.979 (95 % CI 0.973, 0.985), p < 0.000. Substantial agreement was noted with meropenem κ = 0.724 (95 % CI 0.573, 0.875), p < 0.000 while moderate agreement was noted with amoxicillin-clavulanate κ = 0.581 (95 % CI 0.567, 0.595), p < 0.000 and cefepime κ = 0.600 (95 % CI 0.578, 0.622), p < 0.000. Fair agreement was seen with nitrofurantoin κ = 0.351 (95 % CI 0.314, 0.388), p < 0.000 while poor agreement was noted with amikacin κ = 0.112 (95 % CI 0.079, 0.145), p < 0.000.

Of the 1103 *S. aureus* MICs analyzed, susceptibility to penicillin, oxacillin, levofloxacin, linezolid, trimethoprim–sulfamethoxazole, vancomycin, clindamycin, erythromycin and tetracycline was comparable between the two guidelines. The susceptibilities, concordance and kappa statistics between the two guidelines are shown in Table 2.

**Table 2:** Susceptibilities of *S.aureus* to various antibiotics and the concordance and kappa statistics between CLSI 2015 and EUCAST 2015 guidelines

Antibiotic	CLSI (%); n = 1103			EUCAST (%); n = 1103			Concordance (%)	Kappa, κ (95 % CI)
	S	I	R	S	I	R		
Penicillin	10.6	0	89.4	10.6	0	89.4	100	1
TMP/SMX	0	0	100	0	0	100	100	1
Clindamycin	98.0	0.3	1.7	98.0	0.1	1.9	99.5	0.904 (0.826, 0.982)
Erythromycin	85.6	0.5	13.9	86.0	0	14.0	99.4	0.978 (0.960, 0.996)
Gentamicin	96.3	0.5	3.2	91.5	0	8.5	94.6	0.537 (0.441, 0.633)
Levofloxacin	90.8	0.3	8.9	90.8	0.3	8.9	100	1
Linezolid	100	0	0	100	0	0	100	1
Oxacillin	93.3	0	6.7	93.3	0	6.7	100	1
Tetracycline	83.2	0.1	16.7	82.4	0.8	16.8	98.9	0.962 (0.940, 0.984)
Vancomycin	100	0	0	100	0	0	100	1

*TMP/SMX* trimethoprim–sulfamethoxazole, *S* susceptible, *I* intermediate, *R* resistant

For *S. aureus* the susceptibilities are generally very similar between the two guidelines. Year by year analysis did not show any differences in trends and susceptibility patterns. The kappa analysis for CLSI 2015 and EUCAST 2015 guidelines revealed perfect agreement for levofloxacin, linezolid, vancomycin, oxacillin, penicillin and trimethoprim–sulfamethoxazole with a kappa statistic of 1 (p < 0.000). An almost perfect agreement was noted with clindamycin κ = 0.904 (95 % CI 0.826, 0.982), p < 0.000, erythromycin κ = 0.978 (95 % CI 0.960, 0.996), p < 0.000 and tetracycline κ = 0.962 (95 % CI 0.940, 0.984), p < 0.000. Moderate agreement was noted with gentamicin κ = 0.537 (95 % CI 0.441, 0.633), p < 0.000.

For the 532 *P. aeruginosa* analyzed there were similar susceptibility patterns noted between CLSI 2015 and EUCAST 2015. Antibiotics analyzed included amikacin, ceftazidime, ciprofloxacin, cefepime, gentamicin, meropenem and piperacillin–tazobactum. The susceptibilities, concordance and kappa statistics between the two guidelines are shown in Table 3. Year by year analysis did not show any differences in trends and susceptibility patterns. The kappa analysis for CLSI 2015 and EUCAST 2015 guidelines revealed moderate to almost perfect agreement for all the anti-pseudomonal antibiotics. P values for the kappa statistics for all the antibiotics were statistically significant at p < 0.000. Overall, the antibiotic susceptibility patterns were quite similar regardless of whether CLSI or EUCAST 2015 guidelines were used.

**Table 3:** Susceptibilities of *P.aeruginosa* to various antibiotics and the concordance and kappa statistics between CLSI 2015 and EUCAST 2015 guidelines

	CLSI (%); n = 532			EUCAST (%); n = 532			Concordance (%)	Kappa, κ (95 % CI)
	S	I	R	S	I	R		
Amikacin	79.5	3.0	17.5	72.2	7.3	20.5	89.7	0.734 (0.703; 0.765)
Ceftazidime	70.9	4.7	24.4	70.9	0	29.1	95.3	0.890 (0.870; 0.910)
Cefepime	72.6	4.7	22.7	72.6	0	27.4	95.3	0.886 (0.866; 0.906)
Gentamicin	72.6	6.2	21.2	72.6	0	27.4	93.8	0.851 (0.829; 0.873)
Meropenem	64.1	8.3	27.6	64.1	12.8	23.1	95.5	0.912 (0.895; 0.929)
Pip-taz	64.5	8.3	27.3	64.5	0	35.5	91.7	0.830 (0.808; 0.852)
Ciprofloxacin	71.8	5.3	22.9	66.2	5.6	28.2	89.1	0.762 (0.736; 0.788)

*Pip-Taz* piperacillin–tazobactum, *CI* confidence interval, *S* susceptible, *I* intermediate, *R* resistant

## Discussion

The morbidity and mortality associated with communicable diseases including bacterial infections is quite significant in developing countries [10]. Antibiotics play a critical role in treating such infections especially when instituted in a timely fashion more so when the bacteria is susceptible to the antibiotic given. The determination of accurate antibiotic susceptibility is therefore an important cog in the clinical care of bacterial infections especially in organisms that possess acquired resistance mechanisms and careful consideration should be given when deciding how to interpret phenotypic susceptibility data [11]. In Kenya, many laboratories have adopted CLSI guidelines as a basis for interpreting their susceptibility data despite the fact that it costs between US $300–$500. These guidelines are updated annually and therefore require laboratories to keep on purchasing them at a cost that is prohibitive to most public laboratories. Failure to stay updated may result in misinterpretation of susceptibility. For example in 2013, CLSI abandoned the use of oxacillin disc diffusion in determining whether a *S. aureus* isolate is methicillin resistant in favour of cefoxitin because it was more accurate in determining the presence of a *mecA* mediated mechanism of resistance [12]. In 2012, it adopted a new disc diffusion susceptibility cut off for ciprofloxacin of 31 mm up from 21 mm for non-typhoidal *Salmonella* (NTS) isolated from invasive specimens and all *Salmonella* Typhi isolated from both invasive and non-invasive specimens. The MIC for the same was reduced from 1 to 0.06 μg/mL resulting in many *Salmonella* spp. previously reported as susceptible to fall into the intermediate category, and many that were intermediate were now categorized as resistant [13]. In 2013, CLSI recommended that these cut offs should apply to all *Salmonella* spp. including NTS from non-invasive specimens [12, 14]. In the same year new levofloxacin and ofloxacin breakpoints were introduced for *Salmonella* spp. including *Salmonella* Typhi. These changes were motivated by an increased risk of treatment failure in patients with decreased ciprofloxacin susceptible *Salmonella spp* [15]. In 2012 again, CLSI reduced meropenem and piperacillin-tazobactum MIC breakpoints for *P. aeruginosa* from ≤4 to ≤2 ug/mL and ≤64 ug/mL to ≤16 ug/mL respectively [13]. These examples highlight the importance of remaining up-to-date and emphasizes the need for an affordable, up-to-date and readily available guideline for the interpretation of antibiotic susceptibility.

In our comparison of CLSI and EUCAST guidelines for interpretation of antibiotic susceptibility, for *E. coli*, most of the antibiotics had moderate to perfect agreement between the two guidelines with kappa values ranging from 0.581 to 1 and two-thirds of them having almost perfect or perfect agreement with kappa values ranging from 0.859 to 1. Poor agreement was noted with amikacin with EUCAST having a more stringent breakpoint for susceptibility of ≤8 mg/L compared to the CLSI breakpoint of ≤16 mg/L. The major discrepancy was in the intermediate and susceptible categories as 8.8 % of isolates labeled as intermediate by EUCAST were all categorized as susceptible by CLSI. EUCAST guidelines eliminated the intermediate category for some antibiotics and this explains the reduced level of agreement for some of the antibiotics. For example, CLSI categorized 23.8 % of *E. coli* as having intermediate susceptibility to amoxicillin-clavulanate but were all categorized as resistant by EUCAST while 11.9 % categorized as intermediate susceptibility to nitrofurantoin by CLSI were categorized as susceptible by EUCAST. From a clinical stand point, reclassifying amoxicillin-clavulanate from intermediate to resistant is unlikely to adversely affect the patient as it simply removes it from being a therapeutic consideration. As for nitrofurantoin, its ability to concentrate in urine enables it to achieve significant concentrations and eliminate isolates that may have intermediate susceptibility. Therefore, the reclassification of isolates that are intermediate by CLSI to susceptible by EUCAST is unlikely to contribute to adverse outcomes for patients with urinary tract infections. The EUCAST guidelines have slightly more stringent breakpoints for some antibiotics in an effort to curb the inappropriate use of antibiotics and control the rising rates of antibiotic resistance.

For *S. aureus*, all except one of the antibiotics had moderate to perfect agreement and three quarters of them had almost perfect or perfect agreement with kappa values ranging from 0.862 to 1. The two guidelines performed equally in the detection of the rate of methicillin resistant *Staphylococcus aureus* (MRSA) of 7.2 %. Vancomycin susceptible *Staphylococcus aureus* (VSSA) were also detected equally by the two guidelines. EUCAST eliminated the intermediate category for vancomycin in a bid to discourage the reporting of Glycopeptide-intermediate *Staphylococcus aureus* (GISA) due to poor response even to increased doses of vancomycin [8, 16]. In view of the fact that no vancomycin resistant *Staphylococcus aureus* (VRSA) has been identified in our set up as yet, these changes are unlikely to influence interpretation of breakpoints. The moderate level of agreement for gentamicin is due to the more stringent breakpoints by EUCAST leading to a much higher resistance rate of 8.6 % compared to 3.2 % by CLSI. The difference between the two susceptibility cut-offs is two dilutions and this may require further harmonization. Gentamicin is rarely used as monotherapy in treating gram positive bacteria and as such this difference in MIC cut off is unlikely to be clinically significant.

For *P. aeruginosa*, five out of seven of the antibiotics had almost perfect agreement with kappa values ranging from 0.830 to 0.912 with the remaining two having moderate agreement with kappa values of 0.762 and 0.734. EUCAST abolished the intermediate category for ceftazidime, cefepime, gentamicin and piperacillin-tazobactum reclassifying the MICs as resistant. This accounts for the slightly reduced level of agreement. For amikacin and ciprofloxacin, with moderate agreement, EUCAST has more stringent breakpoints for susceptibility of ≤8 and ≤0.5 ug/mL compared to CLSI breakpoints of ≤16 and ≤1 ug/mL respectively. These accounted for the reduced level of agreement and may require further harmonization between the two breakpoints. For meropenem, EUCAST uses a resistant breakpoint of >8 ug/mL while CLSI uses ≥8 ug/mL. In effect this has led to a slightly higher meropenem resistance of 27.6 % compared to 23.1 % for CLSI and EUCAST respectively. This is another aspect that will require harmonization.

Our study is limited by the fact that we only compared the susceptibility for three bacteria whose results may not necessarily be generalizable to all clinically relevant gram positive and negative bacteria. However, these three bacteria represent a significant proportion of common bacterial pathogens both in the community and healthcare settings namely *Enterobacteriaceae,* non-fermenting gram negative bacteria and *Staphylococci*. The results obtained are also limited to MICs generated by an automated bacterial identification system which is not widely used in developing countries. For *P. aeruginosa*, Colistin had not yet been included in the gram negative AST cards being used at the time the study was being conducted. Colistin Etest^®^ (bioMerieux, Durham, NC, USA) was only run for multi-drug resistant isolates of clinical significance but this data was not available for analysis. In most laboratories in sub-Saharan Africa, disk diffusion is the preferred mode of antibiotic susceptibility testing. However, disk diffusion cut offs generally approximate MIC cut offs fairly well and we think a similar comparison based on disc diffusion cut offs would yield similar results.

## Conclusion

Our study shows acceptable level of agreement between EUCAST and CLSI 2015 AST guidelines for *E. coli, S. aureus* and *P. aeruginosa* and laboratories with similar antibiotic susceptibility patterns may choose to adopt either guideline without fear of significantly altering reported antibiotic susceptibility. With EUCAST guidelines being freely available it should be considered as an alternative especially in resource poor settings in order to maintain up-to-date antibiotic susceptibility interpretation.

## Abbreviations

AKUHN: Aga Khan University Hospital, Nairobi
Amox-Clav: amoxicillin–clavulanate
AST: antimicrobial susceptibility testing
CLSI: Clinical Laboratory Standards Institute
ECOFFS: epidemiological cut-offs
ESBL: extended spectrum beta lactamase
Etest: epsilometer test
EUCAST: European Committee on Antimicrobial Susceptibility Testing
*E. coli*: *Escherichia coli*
FDA: Food and Drug Administration
GC: General Committee
GISA: Glycopeptide-intermediate *Staphylococcus aureus*
IBM: International Business Machines Corporation (Armonk, New York, United States of America)
MIC: minimum inhibitory concentration
MRSA: methicillin resistant *Staphylococcus aureus*
NAC: National Antimicrobial Susceptibility Testing Committees
NTS: non-typhoidal salmonella
Pip-Taz: piperacillin–tazobactum
PK-PD: pharmacokinetic–pharmacodynamics
SPSS: statistical package for the social sciences
*S. aureus*: *Staphylococcus aureus*
TMP/SMX: trimethoprim–sulfamethoxazole
US $: United States of America dollar
VRSA: vancomycin resistant *Staphylococcus aureus*
VSSA: vancomycin susceptible *Staphylococcus aureus*
